# A novel and simple method for identifying the lung intersegmental plane with an infrared thermography

**DOI:** 10.1186/1749-8090-10-S1-A113

**Published:** 2015-12-16

**Authors:** Kei Sakamoto, Masato Kanzaki, Akira Ogihara, Shota Mitsuboshi, Takehito Yoshiya, Takuma Kikkawa, Tamami Isaka, Kunihiro Oyama, Masahide Murasugi, Takamasa Onuki

**Affiliations:** 1 Department of Surgery, Tokyo Women's Medical University, Shinjuku, Tokyo, 162-8666, Japan

## Background/Introduction

In performing lung segmentectomy, identification of the intersegmental plane is a very important process. To identify the intersegmental plane, several methods have been reported: (1) making a border line between inflated and deflated segments; (2) using infrared thoracoscopy with intravenous or intrabronchial injection of indocyanine green; (3) identifying intersegmental pulmonary vein using 3-dimensional pulmonary models. However each method has its own problems, and the choice of these methods is controversial. Recently, in ex-vivo study, the relation between lung perfusion and lung surface temperature was reported, whereas the change of surface temperature during lung segmentectomy has not been clarified.

## Aims/Objectives

We hypothesized that surface temperatures of resecting segments or lobes decrease due to blood flow suppression by ligation of target arteries and veins. Therefore we tried to identify the intersegmental or interlober plane with an infrared thermography.

## Method

We used experimental pig models, and performed lung lobectomies (n = 2), and segmentectomies (n = 4). During surgical procedure, lung surface temperatures were monitored with an infrared thermography through the thoracotomies.

## Results

After ligation of target arteries and veins, differences in temperature between resecting and preserving areas were clearly identified, and transitional zones of surface temperature were visualized as sharp lines. And intersegmental or interlober planes detected by an infrared thermography were exactly matched with the lines detected by inflate-deflate lines.

## Discussion/Conclusion

Result of this study shows the possibility of the visualization of intersegmental or interlober planes with an infrared thermography. The thermographical method is noninvasive, and we just need to monitor the lung surface with an infrared thermography. In addition we can use the thermographical method together with conventional methods during operation. We consider that the thermographical method can help thoracic surgeons who perform lung segmentectomy to identify the intersegmental plane simply and precisely.

